# Supplementary material for: Fish perform like mammals and birds in inhibitory motor control tasks
Source: Sci Rep. 2017 Oct 13;7:13144. doi: 10.1038/s41598-017-13447-4 (PMC5640690; doi:10.1038/s41598-017-13447-4)
Supplement: Supplementary file 1 — Dataset 1 [file 41598_2017_13447_MOESM1_ESM.doc]

**Fish perform like mammals and birds in inhibitory motor control tasks**

Tyrone Lucon-Xiccato, Elia Gatto, & Angelo Bisazza

^*^Correspondence: tyrone.luconxiccato@unipd.it

**Supplementary Information: Data of experiment 1 and experiment 2**

Experiment 1

| ID | trial | time | score |
| --- | --- | --- | --- |
| 1 | 1 | 15 | correct |
| 1 | 2 | 18 | correct |
| 1 | 3 | 7 | correct |
| 1 | 4 | 17 | correct |
| 1 | 5 | 13 | correct |
| 1 | 6 | 8 | correct |
| 1 | 7 | 14 | correct |
| 1 | 8 | 27 | incorrect |
| 1 | 9 | 7 | correct |
| 1 | 10 | 12 | correct |
| 1 | 11 | 6 | correct |
| 1 | 12 | 7 | correct |
| 1 | 13 | 17 | incorrect |
| 1 | 14 | 11 | correct |
| 1 | 15 | 4 | correct |
| 1 | 16 | 2 | correct |
| 1 | 17 | 5 | correct |
| 1 | 18 | 15 | incorrect |
| 1 | 19 | 5 | correct |
| 1 | 20 | 3 | correct |
| 1 | 21 | 17 | incorrect |
| 1 | 22 | 5 | correct |
| 1 | 23 | 6 | correct |
| 1 | 24 | 4 | correct |
| 1 | 25 | 8 | incorrect |
| 1 | 26 | 3 | correct |
| 1 | 27 | 18 | incorrect |
| 1 | 28 | 6 | correct |
| 1 | 29 | 11 | incorrect |
| 1 | 30 | 24 | incorrect |
| 1 | 31 | 5 | correct |
| 1 | 32 | 5 | correct |
| 1 | 33 | 7 | incorrect |
| 1 | 34 | 9 | correct |
| 1 | 35 | 6 | correct |
| 1 | 36 | 6 | correct |
| 1 | 37 | 13 | incorrect |
| 1 | 38 | 2 | correct |
| 1 | 39 | 3 | correct |
| 1 | 40 | 9 | correct |
| 1 | 41 | 3 | correct |
| 1 | 42 | 5 | correct |
| 1 | 43 | 5 | correct |
| 1 | 44 | 57 | incorrect |
| 1 | 45 | 3 | correct |
| 1 | 46 | 3 | incorrect |
| 1 | 47 | 10 | incorrect |
| 1 | 48 | 11 | incorrect |
| 1 | 49 | 3 | correct |
| 1 | 50 | 4 | correct |
| 2 | 1 | 46 | incorrect |
| 2 | 2 | 12 | incorrect |
| 2 | 3 | 54 | correct |
| 2 | 4 | 355 | incorrect |
| 2 | 5 | 900 | incorrect |
| 2 | 6 | 25 | correct |
| 2 | 7 | 26 | correct |
| 2 | 8 | 55 | incorrect |
| 2 | 9 | 401 | incorrect |
| 2 | 10 | 115 | correct |
| 2 | 11 | 76 | incorrect |
| 2 | 12 | 11 | correct |
| 2 | 13 | 10 | incorrect |
| 2 | 14 | 10 | correct |
| 2 | 15 | 20 | correct |
| 2 | 16 | 269 | incorrect |
| 2 | 17 | 16 | incorrect |
| 2 | 18 | 56 | correct |
| 2 | 19 | 251 | correct |
| 2 | 20 | 176 | incorrect |
| 2 | 21 | 32 | incorrect |
| 2 | 22 | 263 | incorrect |
| 2 | 23 | 34 | incorrect |
| 2 | 24 | 50 | incorrect |
| 2 | 25 | 27 | correct |
| 2 | 26 | 10 | correct |
| 2 | 27 | 201 | correct |
| 2 | 28 | 10 | correct |
| 2 | 29 | 66 | correct |
| 2 | 30 | 364 | incorrect |
| 2 | 31 | 178 | correct |
| 2 | 32 | 82 | correct |
| 2 | 33 | 561 | incorrect |
| 2 | 34 | 120 | correct |
| 2 | 35 | 108 | incorrect |
| 2 | 36 | 114 | correct |
| 2 | 37 | 55 | correct |
| 2 | 38 | 71 | correct |
| 2 | 39 | 25 | correct |
| 2 | 40 | 41 | correct |
| 2 | 41 | 36 | incorrect |
| 2 | 42 | 16 | correct |
| 2 | 43 | 16 | incorrect |
| 2 | 44 | 21 | correct |
| 2 | 45 | 35 | correct |
| 2 | 46 | 25 | correct |
| 2 | 47 | 33 | correct |
| 2 | 48 | 57 | incorrect |
| 2 | 49 | 32 | correct |
| 2 | 50 | 18 | correct |
| 3 | 1 | 39 | correct |
| 3 | 2 | 73 | incorrect |
| 3 | 3 | 19 | correct |
| 3 | 4 | 30 | correct |
| 3 | 5 | 5 | correct |
| 3 | 6 | 68 | incorrect |
| 3 | 7 | 10 | correct |
| 3 | 8 | 80 | incorrect |
| 3 | 9 | 93 | correct |
| 3 | 10 | 172 | correct |
| 3 | 11 | 150 | incorrect |
| 3 | 12 | 24 | correct |
| 3 | 13 | 54 | correct |
| 3 | 14 | 599 | incorrect |
| 3 | 15 | 45 | correct |
| 3 | 16 | 158 | incorrect |
| 3 | 17 | 6 | correct |
| 3 | 18 | 19 | correct |
| 3 | 19 | 88 | incorrect |
| 3 | 20 | 900 | incorrect |
| 3 | 21 | 61 | incorrect |
| 3 | 22 | 81 | correct |
| 3 | 23 | 7 | correct |
| 3 | 24 | 900 | incorrect |
| 3 | 25 | 900 | incorrect |
| 3 | 26 | 30 | correct |
| 3 | 27 | 25 | correct |
| 3 | 28 | 6 | correct |
| 3 | 29 | 64 | correct |
| 3 | 30 | 51 | incorrect |
| 3 | 31 | 23 | correct |
| 3 | 32 | 6 | incorrect |
| 3 | 33 | 20 | correct |
| 3 | 34 | 24 | correct |
| 3 | 35 | 834 | incorrect |
| 3 | 36 | 21 | incorrect |
| 3 | 37 | 21 | correct |
| 3 | 38 | 3 | correct |
| 3 | 39 | 4 | incorrect |
| 3 | 40 | 8 | incorrect |
| 3 | 41 | 52 | incorrect |
| 3 | 42 | 21 | incorrect |
| 3 | 43 | 5 | correct |
| 3 | 44 | 5 | correct |
| 3 | 45 | 4 | correct |
| 3 | 46 | 28 | correct |
| 3 | 47 | 9 | correct |
| 3 | 48 | 25 | incorrect |
| 3 | 49 | 29 | correct |
| 3 | 50 | 32 | incorrect |
| 4 | 1 | 23 | incorrect |
| 4 | 2 | 30 | correct |
| 4 | 3 | 71 | incorrect |
| 4 | 4 | 103 | incorrect |
| 4 | 5 | 140 | incorrect |
| 4 | 6 | 40 | correct |
| 4 | 7 | 20 | correct |
| 4 | 8 | 19 | correct |
| 4 | 9 | 67 | correct |
| 4 | 10 | 49 | correct |
| 4 | 11 | 12 | incorrect |
| 4 | 12 | 25 | incorrect |
| 4 | 13 | 66 | correct |
| 4 | 14 | 23 | correct |
| 4 | 15 | 9 | correct |
| 4 | 16 | 13 | correct |
| 4 | 17 | 23 | incorrect |
| 4 | 18 | 7 | correct |
| 4 | 19 | 28 | correct |
| 4 | 20 | 4 | correct |
| 4 | 21 | 24 | incorrect |
| 4 | 22 | 17 | correct |
| 4 | 23 | 32 | incorrect |
| 4 | 24 | 3 | correct |
| 4 | 25 | 8 | incorrect |
| 4 | 26 | 7 | incorrect |
| 4 | 27 | 5 | correct |
| 4 | 28 | 9 | correct |
| 4 | 29 | 22 | correct |
| 4 | 30 | 4 | correct |
| 4 | 31 | 9 | correct |
| 4 | 32 | 13 | incorrect |
| 4 | 33 | 3 | correct |
| 4 | 34 | 200 | incorrect |
| 4 | 35 | 9 | correct |
| 4 | 36 | 12 | correct |
| 4 | 37 | 22 | correct |
| 4 | 38 | 5 | correct |
| 4 | 39 | 2 | correct |
| 4 | 40 | 182 | incorrect |
| 4 | 41 | 4 | correct |
| 4 | 42 | 5 | correct |
| 4 | 43 | 19 | correct |
| 4 | 44 | 25 | correct |
| 4 | 45 | 11 | correct |
| 4 | 46 | 16 | correct |
| 4 | 47 | 36 | correct |
| 4 | 48 | 230 | correct |
| 4 | 49 | 26 | correct |
| 4 | 50 | 63 | correct |
| 5 | 1 | 23 | correct |
| 5 | 2 | 5 | correct |
| 5 | 3 | 14 | correct |
| 5 | 4 | 6 | correct |
| 5 | 5 | 8 | correct |
| 5 | 6 | 42 | incorrect |
| 5 | 7 | 19 | correct |
| 5 | 8 | 23 | correct |
| 5 | 9 | 9 | correct |
| 5 | 10 | 17 | correct |
| 5 | 11 | 48 | incorrect |
| 5 | 12 | 18 | correct |
| 5 | 13 | 11 | correct |
| 5 | 14 | 13 | correct |
| 5 | 15 | 9 | incorrect |
| 5 | 16 | 25 | correct |
| 5 | 17 | 34 | incorrect |
| 5 | 18 | 8 | correct |
| 5 | 19 | 6 | correct |
| 5 | 20 | 7 | correct |
| 5 | 21 | 73 | incorrect |
| 5 | 22 | 10 | correct |
| 5 | 23 | 9 | correct |
| 5 | 24 | 7 | correct |
| 5 | 25 | 8 | correct |
| 5 | 26 | 14 | incorrect |
| 5 | 27 | 6 | correct |
| 5 | 28 | 5 | correct |
| 5 | 29 | 17 | incorrect |
| 5 | 30 | 5 | correct |
| 5 | 31 | 13 | incorrect |
| 5 | 32 | 15 | correct |
| 5 | 33 | 8 | correct |
| 5 | 34 | 71 | incorrect |
| 5 | 35 | 6 | correct |
| 5 | 36 | 48 | incorrect |
| 5 | 37 | 29 | correct |
| 5 | 38 | 12 | incorrect |
| 5 | 39 | 9 | correct |
| 5 | 40 | 21 | incorrect |
| 5 | 41 | 6 | correct |
| 5 | 42 | 23 | correct |
| 5 | 43 | 5 | correct |
| 5 | 44 | 5 | correct |
| 5 | 45 | 24 | incorrect |
| 5 | 46 | 33 | incorrect |
| 5 | 47 | 12 | incorrect |
| 5 | 48 | 10 | incorrect |
| 5 | 49 | 38 | incorrect |
| 5 | 50 | 23 | correct |
| 6 | 1 | 5 | incorrect |
| 6 | 2 | 31 | correct |
| 6 | 3 | 16 | incorrect |
| 6 | 4 | 43 | correct |
| 6 | 5 | 8 | incorrect |
| 6 | 6 | 4 | correct |
| 6 | 7 | 33 | incorrect |
| 6 | 8 | 5 | incorrect |
| 6 | 9 | 5 | correct |
| 6 | 10 | 6 | incorrect |
| 6 | 11 | 48 | incorrect |
| 6 | 12 | 17 | incorrect |
| 6 | 13 | 26 | incorrect |
| 6 | 14 | 12 | correct |
| 6 | 15 | 6 | correct |
| 6 | 16 | 16 | correct |
| 6 | 17 | 6 | correct |
| 6 | 18 | 36 | incorrect |
| 6 | 19 | 10 | incorrect |
| 6 | 20 | 8 | correct |
| 6 | 21 | 12 | correct |
| 6 | 22 | 20 | incorrect |
| 6 | 23 | 3 | correct |
| 6 | 24 | 16 | correct |
| 6 | 25 | 10 | correct |
| 6 | 26 | 15 | correct |
| 6 | 27 | 5 | correct |
| 6 | 28 | 14 | incorrect |
| 6 | 29 | 6 | incorrect |
| 6 | 30 | 4 | correct |
| 6 | 31 | 16 | incorrect |
| 6 | 32 | 4 | incorrect |
| 6 | 33 | 23 | incorrect |
| 6 | 34 | 1 | correct |
| 6 | 35 | 5 | correct |
| 6 | 36 | 7 | incorrect |
| 6 | 37 | 4 | incorrect |
| 6 | 38 | 5 | correct |
| 6 | 39 | 14 | incorrect |
| 6 | 40 | 16 | incorrect |
| 6 | 41 | 7 | correct |
| 6 | 42 | 14 | incorrect |
| 6 | 43 | 52 | correct |
| 6 | 44 | 6 | correct |
| 6 | 45 | 19 | correct |
| 6 | 46 | 10 | correct |
| 6 | 47 | 15 | correct |
| 6 | 48 | 133 | correct |
| 6 | 49 | 59 | correct |
| 6 | 50 | 4 | correct |
| 7 | 1 | 35 | correct |
| 7 | 2 | 76 | incorrect |
| 7 | 3 | 21 | correct |
| 7 | 4 | 32 | correct |
| 7 | 5 | 10 | correct |
| 7 | 6 | 16 | correct |
| 7 | 7 | 11 | incorrect |
| 7 | 8 | 5 | correct |
| 7 | 9 | 15 | correct |
| 7 | 10 | 8 | correct |
| 7 | 11 | 8 | correct |
| 7 | 12 | 10 | correct |
| 7 | 13 | 10 | correct |
| 7 | 14 | 3 | correct |
| 7 | 15 | 4 | correct |
| 7 | 16 | 15 | incorrect |
| 7 | 17 | 10 | correct |
| 7 | 18 | 8 | correct |
| 7 | 19 | 7 | correct |
| 7 | 20 | 24 | incorrect |
| 7 | 21 | 33 | correct |
| 7 | 22 | 4 | correct |
| 7 | 23 | 14 | correct |
| 7 | 24 | 7 | correct |
| 7 | 25 | 14 | correct |
| 7 | 26 | 10 | correct |
| 7 | 27 | 17 | incorrect |
| 7 | 28 | 12 | incorrect |
| 7 | 29 | 16 | incorrect |
| 7 | 30 | 33 | incorrect |
| 7 | 31 | 10 | incorrect |
| 7 | 32 | 7 | correct |
| 7 | 33 | 7 | incorrect |
| 7 | 34 | 4 | correct |
| 7 | 35 | 13 | correct |
| 7 | 36 | 17 | correct |
| 7 | 37 | 5 | incorrect |
| 7 | 38 | 8 | incorrect |
| 7 | 39 | 7 | incorrect |
| 7 | 40 | 15 | incorrect |
| 7 | 41 | 2 | correct |
| 7 | 42 | 4 | correct |
| 7 | 43 | 40 | incorrect |
| 7 | 44 | 8 | correct |
| 7 | 45 | 179 | correct |
| 7 | 46 | 22 | correct |
| 7 | 47 | 8 | correct |
| 7 | 48 | 12 | incorrect |
| 7 | 49 | 5 | incorrect |
| 7 | 50 | 5 | correct |
| 8 | 1 | 111 | incorrect |
| 8 | 2 | 45 | incorrect |
| 8 | 3 | 25 | correct |
| 8 | 4 | 40 | incorrect |
| 8 | 5 | 8 | incorrect |
| 8 | 6 | 16 | incorrect |
| 8 | 7 | 14 | incorrect |
| 8 | 8 | 12 | incorrect |
| 8 | 9 | 14 | incorrect |
| 8 | 10 | 10 | incorrect |
| 8 | 11 | 18 | incorrect |
| 8 | 12 | 4 | correct |
| 8 | 13 | 5 | correct |
| 8 | 14 | 9 | correct |
| 8 | 15 | 7 | correct |
| 8 | 16 | 34 | incorrect |
| 8 | 17 | 58 | incorrect |
| 8 | 18 | 13 | incorrect |
| 8 | 19 | 28 | incorrect |
| 8 | 20 | 14 | correct |
| 8 | 21 | 9 | correct |
| 8 | 22 | 5 | incorrect |
| 8 | 23 | 7 | correct |
| 8 | 24 | 20 | incorrect |
| 8 | 25 | 10 | correct |
| 8 | 26 | 13 | correct |
| 8 | 27 | 10 | incorrect |
| 8 | 28 | 7 | correct |
| 8 | 29 | 12 | incorrect |
| 8 | 30 | 11 | incorrect |
| 8 | 31 | 10 | correct |
| 8 | 32 | 12 | incorrect |
| 8 | 33 | 13 | correct |
| 8 | 34 | 20 | incorrect |
| 8 | 35 | 12 | incorrect |
| 8 | 36 | 13 | correct |
| 8 | 37 | 9 | correct |
| 8 | 38 | 7 | correct |
| 8 | 39 | 3 | correct |
| 8 | 40 | 9 | incorrect |
| 8 | 41 | 10 | correct |
| 8 | 42 | 7 | correct |
| 8 | 43 | 20 | incorrect |
| 8 | 44 | 20 | incorrect |
| 8 | 45 | 11 | correct |
| 8 | 46 | 12 | incorrect |
| 8 | 47 | 3 | correct |
| 8 | 48 | 5 | correct |
| 8 | 49 | 14 | incorrect |
| 8 | 50 | 9 | correct |
| 9 | 1 | 41 | incorrect |
| 9 | 2 | 31 | incorrect |
| 9 | 3 | 19 | incorrect |
| 9 | 4 | 10 | incorrect |
| 9 | 5 | 35 | correct |
| 9 | 6 | 7 | incorrect |
| 9 | 7 | 16 | incorrect |
| 9 | 8 | 18 | incorrect |
| 9 | 9 | 16 | incorrect |
| 9 | 10 | 16 | incorrect |
| 9 | 11 | 27 | incorrect |
| 9 | 12 | 3 | incorrect |
| 9 | 13 | 5 | correct |
| 9 | 14 | 10 | incorrect |
| 9 | 15 | 8 | correct |
| 9 | 16 | 4 | correct |
| 9 | 17 | 8 | correct |
| 9 | 18 | 5 | incorrect |
| 9 | 19 | 7 | correct |
| 9 | 20 | 8 | correct |
| 9 | 21 | 11 | incorrect |
| 9 | 22 | 10 | incorrect |
| 9 | 23 | 25 | correct |
| 9 | 24 | 12 | incorrect |
| 9 | 25 | 10 | correct |
| 9 | 26 | 7 | incorrect |
| 9 | 27 | 8 | incorrect |
| 9 | 28 | 111 | incorrect |
| 9 | 29 | 29 | incorrect |
| 9 | 30 | 11 | correct |
| 9 | 31 | 24 | correct |
| 9 | 32 | 5 | correct |
| 9 | 33 | 4 | correct |
| 9 | 34 | 6 | incorrect |
| 9 | 35 | 900 | incorrect |
| 9 | 36 | 5 | correct |
| 9 | 37 | 9 | correct |
| 9 | 38 | 6 | incorrect |
| 9 | 39 | 5 | correct |
| 9 | 40 | 4 | correct |
| 9 | 41 | 5 | incorrect |
| 9 | 42 | 11 | correct |
| 9 | 43 | 9 | incorrect |
| 9 | 44 | 24 | incorrect |
| 9 | 45 | 22 | incorrect |
| 9 | 46 | 8 | incorrect |
| 9 | 47 | 8 | incorrect |
| 9 | 48 | 82 | incorrect |
| 9 | 49 | 18 | correct |
| 9 | 50 | 10 | incorrect |
| 10 | 1 | 11 | correct |
| 10 | 2 | 10 | incorrect |
| 10 | 3 | 8 | correct |
| 10 | 4 | 12 | incorrect |
| 10 | 5 | 18 | incorrect |
| 10 | 6 | 17 | correct |
| 10 | 7 | 12 | correct |
| 10 | 8 | 16 | incorrect |
| 10 | 9 | 4 | incorrect |
| 10 | 10 | 9 | incorrect |
| 10 | 11 | 23 | incorrect |
| 10 | 12 | 6 | incorrect |
| 10 | 13 | 7 | correct |
| 10 | 14 | 3 | incorrect |
| 10 | 15 | 8 | correct |
| 10 | 16 | 27 | incorrect |
| 10 | 17 | 13 | correct |
| 10 | 18 | 7 | correct |
| 10 | 19 | 8 | correct |
| 10 | 20 | 23 | correct |
| 10 | 21 | 20 | incorrect |
| 10 | 22 | 10 | correct |
| 10 | 23 | 9 | correct |
| 10 | 24 | 13 | incorrect |
| 10 | 25 | 7 | correct |
| 10 | 26 | 9 | correct |
| 10 | 27 | 18 | incorrect |
| 10 | 28 | 6 | correct |
| 10 | 29 | 22 | incorrect |
| 10 | 30 | 236 | incorrect |
| 10 | 31 | 19 | incorrect |
| 10 | 32 | 11 | incorrect |
| 10 | 33 | 31 | incorrect |
| 10 | 34 | 40 | incorrect |
| 10 | 35 | 12 | correct |
| 10 | 36 | 3 | correct |
| 10 | 37 | 4 | correct |
| 10 | 38 | 17 | correct |
| 10 | 39 | 20 | correct |
| 10 | 40 | 52 | incorrect |
| 10 | 41 | 7 | incorrect |
| 10 | 42 | 13 | correct |
| 10 | 43 | 9 | correct |
| 10 | 44 | 63 | incorrect |
| 10 | 45 | 7 | incorrect |
| 10 | 46 | 11 | correct |
| 10 | 47 | 15 | correct |
| 10 | 48 | 9 | correct |
| 10 | 49 | 6 | correct |
| 10 | 50 | 68 | Incorrect |

Experiment 2

| ID | trial | time | score |
| --- | --- | --- | --- |
| 1 | 1 | 0 | correct |
| 1 | 2 | 257 | incorrect |
| 1 | 3 | 313 | incorrect |
| 1 | 4 | 29 | incorrect |
| 1 | 5 | 416 | incorrect |
| 1 | 6 | 399 | incorrect |
| 1 | 7 | 173 | incorrect |
| 1 | 8 | 27 | incorrect |
| 1 | 9 | 111 | incorrect |
| 1 | 10 | 62 | incorrect |
| 1 | 11 | 14 | incorrect |
| 1 | 12 | 0 | correct |
| 1 | 13 | 14 | incorrect |
| 1 | 14 | 15 | incorrect |
| 1 | 15 | 0 | correct |
| 1 | 16 | 23 | incorrect |
| 1 | 17 | 3 | incorrect |
| 1 | 18 | 6 | incorrect |
| 1 | 19 | 15 | incorrect |
| 1 | 20 | 87 | incorrect |
| 1 | 21 | 7 | incorrect |
| 1 | 22 | 41 | incorrect |
| 1 | 23 | 136 | incorrect |
| 1 | 24 | 6 | incorrect |
| 1 | 25 | 12 | incorrect |
| 2 | 1 | 11 | incorrect |
| 2 | 2 | 72 | incorrect |
| 2 | 3 | 7 | incorrect |
| 2 | 4 | 12 | incorrect |
| 2 | 5 | 17 | incorrect |
| 2 | 6 | 7 | incorrect |
| 2 | 7 | 21 | incorrect |
| 2 | 8 | 0 | correct |
| 2 | 9 | 7 | incorrect |
| 2 | 10 | 0 | correct |
| 2 | 11 | 9 | incorrect |
| 2 | 12 | 0 | correct |
| 2 | 13 | 0 | correct |
| 2 | 14 | 0 | correct |
| 2 | 15 | 30 | incorrect |
| 2 | 16 | 0 | correct |
| 2 | 17 | 0 | correct |
| 2 | 18 | 0 | correct |
| 2 | 19 | 0 | correct |
| 2 | 20 | 0 | correct |
| 2 | 21 | 3 | incorrect |
| 2 | 22 | 0 | correct |
| 2 | 23 | 0 | correct |
| 2 | 24 | 44 | incorrect |
| 2 | 25 | 0 | correct |
| 3 | 1 | 268 | incorrect |
| 3 | 2 | 194 | incorrect |
| 3 | 3 | 113 | incorrect |
| 3 | 4 | 60 | incorrect |
| 3 | 5 | 0 | correct |
| 3 | 6 | 302 | incorrect |
| 3 | 7 | 58 | incorrect |
| 3 | 8 | 0 | correct |
| 3 | 9 | 22 | incorrect |
| 3 | 10 | 14 | incorrect |
| 3 | 11 | 233 | incorrect |
| 3 | 12 | 55 | incorrect |
| 3 | 13 | 26 | incorrect |
| 3 | 14 | 0 | correct |
| 3 | 15 | 72 | incorrect |
| 3 | 16 | 0 | correct |
| 3 | 17 | 12 | incorrect |
| 3 | 18 | 0 | correct |
| 3 | 19 | 0 | correct |
| 3 | 20 | 0 | correct |
| 3 | 21 | 8 | incorrect |
| 3 | 22 | 27 | incorrect |
| 3 | 23 | 87 | incorrect |
| 3 | 24 | 0 | correct |
| 3 | 25 | 34 | incorrect |
| 4 | 1 | 1 | incorrect |
| 4 | 2 | 13 | incorrect |
| 4 | 3 | 9 | incorrect |
| 4 | 4 | 7 | incorrect |
| 4 | 5 | 0 | correct |
| 4 | 6 | 11 | incorrect |
| 4 | 7 | 9 | incorrect |
| 4 | 8 | 5 | incorrect |
| 4 | 9 | 4 | incorrect |
| 4 | 10 | 0 | correct |
| 4 | 11 | 22 | incorrect |
| 4 | 12 | 0 | correct |
| 4 | 13 | 11 | incorrect |
| 4 | 14 | 13 | incorrect |
| 4 | 15 | 572 | incorrect |
| 4 | 16 | 0 | correct |
| 4 | 17 | 0 | correct |
| 4 | 18 | 0 | correct |
| 4 | 19 | 0 | correct |
| 4 | 20 | 0 | correct |
| 4 | 21 | 6 | incorrect |
| 4 | 22 | 0 | correct |
| 4 | 23 | 0 | correct |
| 4 | 24 | 0 | correct |
| 4 | 25 | 0 | correct |
| 5 | 1 | 37 | incorrect |
| 5 | 2 | 58 | incorrect |
| 5 | 3 | 21 | incorrect |
| 5 | 4 | 36 | incorrect |
| 5 | 5 | 0 | correct |
| 5 | 6 | 17 | incorrect |
| 5 | 7 | 73 | incorrect |
| 5 | 8 | 25 | incorrect |
| 5 | 9 | 34 | incorrect |
| 5 | 10 | 0 | correct |
| 5 | 11 | 0 | correct |
| 5 | 12 | 0 | correct |
| 5 | 13 | 30 | incorrect |
| 5 | 14 | 11 | incorrect |
| 5 | 15 | 11 | incorrect |
| 5 | 16 | 12 | incorrect |
| 5 | 17 | 9 | incorrect |
| 5 | 18 | 11 | incorrect |
| 5 | 19 | 0 | correct |
| 5 | 20 | 0 | correct |
| 5 | 21 | 0 | correct |
| 5 | 22 | 0 | correct |
| 5 | 23 | 0 | correct |
| 5 | 24 | 0 | correct |
| 5 | 25 | 0 | correct |
| 6 | 1 | 31 | incorrect |
| 6 | 2 | 158 | incorrect |
| 6 | 3 | 20 | incorrect |
| 6 | 4 | 215 | incorrect |
| 6 | 5 | 19 | incorrect |
| 6 | 6 | 12 | incorrect |
| 6 | 7 | 7 | incorrect |
| 6 | 8 | 0 | correct |
| 6 | 9 | 0 | correct |
| 6 | 10 | 0 | correct |
| 6 | 11 | 7 | incorrect |
| 6 | 12 | 0 | correct |
| 6 | 13 | 0 | correct |
| 6 | 14 | 0 | correct |
| 6 | 15 | 0 | correct |
| 6 | 16 | 33 | incorrect |
| 6 | 17 | 0 | correct |
| 6 | 18 | 0 | correct |
| 6 | 19 | 0 | correct |
| 6 | 20 | 0 | correct |
| 6 | 21 | 0 | correct |
| 6 | 22 | 491 | incorrect |
| 6 | 23 | 0 | correct |
| 6 | 24 | 17 | incorrect |
| 6 | 25 | 90 | incorrect |
| 7 | 1 | 202 | incorrect |
| 7 | 2 | 30 | incorrect |
| 7 | 3 | 47 | incorrect |
| 7 | 4 | 127 | incorrect |
| 7 | 5 | 0 | correct |
| 7 | 6 | 10 | incorrect |
| 7 | 7 | 596 | incorrect |
| 7 | 8 | 111 | incorrect |
| 7 | 9 | 23 | incorrect |
| 7 | 10 | 58 | incorrect |
| 7 | 11 | 3 | incorrect |
| 7 | 12 | 0 | correct |
| 7 | 13 | 0 | correct |
| 7 | 14 | 185 | incorrect |
| 7 | 15 | 0 | correct |
| 7 | 16 | 0 | correct |
| 7 | 17 | 0 | correct |
| 7 | 18 | 0 | correct |
| 7 | 19 | 7 | incorrect |
| 7 | 20 | 0 | correct |
| 7 | 21 | 0 | correct |
| 7 | 22 | 13 | incorrect |
| 7 | 23 | 0 | correct |
| 7 | 24 | 0 | correct |
| 7 | 25 | 0 | correct |
| 8 | 1 | 0 | correct |
| 8 | 2 | 7 | incorrect |
| 8 | 3 | 0 | correct |
| 8 | 4 | 18 | incorrect |
| 8 | 5 | 22 | incorrect |
| 8 | 6 | 0 | correct |
| 8 | 7 | 4 | incorrect |
| 8 | 8 | 47 | incorrect |
| 8 | 9 | 4 | incorrect |
| 8 | 10 | 0 | correct |
| 8 | 11 | 9 | incorrect |
| 8 | 12 | 7 | incorrect |
| 8 | 13 | 0 | correct |
| 8 | 14 | 9 | incorrect |
| 8 | 15 | 12 | incorrect |
| 8 | 16 | 3 | incorrect |
| 8 | 17 | 4 | incorrect |
| 8 | 18 | 6 | incorrect |
| 8 | 19 | 3 | incorrect |
| 8 | 20 | 2 | incorrect |
| 8 | 21 | 29 | incorrect |
| 8 | 22 | 6 | incorrect |
| 8 | 23 | 0 | correct |
| 8 | 24 | 3 | incorrect |
| 8 | 25 | 14 | incorrect |
| 9 | 1 | 38 | incorrect |
| 9 | 2 | 1 | incorrect |
| 9 | 3 | 0 | correct |
| 9 | 4 | 40 | incorrect |
| 9 | 5 | 113 | incorrect |
| 9 | 6 | 0 | correct |
| 9 | 7 | 119 | incorrect |
| 9 | 8 | 92 | incorrect |
| 9 | 9 | 66 | incorrect |
| 9 | 10 | 0 | correct |
| 9 | 11 | 20 | incorrect |
| 9 | 12 | 15 | incorrect |
| 9 | 13 | 30 | incorrect |
| 9 | 14 | 0 | correct |
| 9 | 15 | 0 | correct |
| 9 | 16 | 118 | incorrect |
| 9 | 17 | 0 | correct |
| 9 | 18 | 0 | correct |
| 9 | 19 | 173 | incorrect |
| 9 | 20 | 392 | incorrect |
| 9 | 21 | 2 | incorrect |
| 9 | 22 | 37 | incorrect |
| 9 | 23 | 138 | incorrect |
| 9 | 24 | 103 | incorrect |
| 9 | 25 | 0 | correct |
| 10 | 1 | 55 | incorrect |
| 10 | 2 | 25 | incorrect |
| 10 | 3 | 3 | incorrect |
| 10 | 4 | 9 | incorrect |
| 10 | 5 | 0 | correct |
| 10 | 6 | 0 | correct |
| 10 | 7 | 0 | correct |
| 10 | 8 | 0 | correct |
| 10 | 9 | 0 | correct |
| 10 | 10 | 0 | correct |
| 10 | 11 | 0 | correct |
| 10 | 12 | 50 | incorrect |
| 10 | 13 | 0 | correct |
| 10 | 14 | 0 | correct |
| 10 | 15 | 0 | correct |
| 10 | 16 | 0 | correct |
| 10 | 17 | 0 | correct |
| 10 | 18 | 23 | incorrect |
| 10 | 19 | 0 | correct |
| 10 | 20 | 0 | correct |
| 10 | 21 | 0 | correct |
| 10 | 22 | 0 | correct |
| 10 | 23 | 0 | correct |
| 10 | 24 | 0 | correct |
| 10 | 25 | 0 | correct |
| 11 | 1 | 26 | incorrect |
| 11 | 2 | 18 | incorrect |
| 11 | 3 | 17 | incorrect |
| 11 | 4 | 4 | incorrect |
| 11 | 5 | 18 | incorrect |
| 11 | 6 | 15 | incorrect |
| 11 | 7 | 106 | incorrect |
| 11 | 8 | 25 | incorrect |
| 11 | 9 | 78 | incorrect |
| 11 | 10 | 117 | incorrect |
| 11 | 11 | 10 | incorrect |
| 11 | 12 | 24 | incorrect |
| 11 | 13 | 32 | incorrect |
| 11 | 14 | 5 | incorrect |
| 11 | 15 | 7 | incorrect |
| 11 | 16 | 7 | incorrect |
| 11 | 17 | 28 | incorrect |
| 11 | 18 | 1 | incorrect |
| 11 | 19 | 130 | incorrect |
| 11 | 20 | 166 | incorrect |
| 11 | 21 | 19 | incorrect |
| 11 | 22 | 4 | incorrect |
| 11 | 23 | 92 | incorrect |
| 11 | 24 | 64 | incorrect |
| 11 | 25 | 92 | incorrect |
| 12 | 1 | 22 | incorrect |
| 12 | 2 | 537 | incorrect |
| 12 | 3 | 122 | incorrect |
| 12 | 4 | 35 | incorrect |
| 12 | 5 | 35 | incorrect |
| 12 | 6 | 99 | incorrect |
| 12 | 7 | 156 | incorrect |
| 12 | 8 | 208 | incorrect |
| 12 | 9 | 102 | incorrect |
| 12 | 10 | 57 | incorrect |
| 12 | 11 | 40 | incorrect |
| 12 | 12 | 26 | incorrect |
| 12 | 13 | 71 | incorrect |
| 12 | 14 | 23 | incorrect |
| 12 | 15 | 175 | incorrect |
| 12 | 16 | 46 | incorrect |
| 12 | 17 | 0 | correct |
| 12 | 18 | 17 | incorrect |
| 12 | 19 | 0 | correct |
| 12 | 20 | 19 | incorrect |
| 12 | 21 | 14 | incorrect |
| 12 | 22 | 127 | incorrect |
| 12 | 23 | 0 | correct |
| 12 | 24 | 81 | incorrect |
| 12 | 25 | 48 | incorrect |
